# Correlation of morphometric properties to meat yield and fatness index in the red strain of the saltwater hard clam Meretrix meretrix

**DOI:** 10.1371/journal.pone.0284730

**Published:** 2023-04-25

**Authors:** Zhidong Zhang, Yangping Wu, Yu Zhang, Yanqing Zhu, Yi Cao, Suhua Chen, Yuheng Peng, Xuefeng Sun, Aihua Chen

**Affiliations:** 1 Marine Fisheries Research Institute of Jiangsu Province, Nantong, Jiangsu Province, China; 2 College of Marine Science and Engineering, Nanjing Normal University, Nanjing, Jiangsu Province, China; 3 National Demonstration Center for Experimental Fisheries Science Education, Shanghai Ocean University, Shanghai, China; Ain Shams University Faculty of Agriculture, EGYPT

## Abstract

To determine the relevance of morphometric properties attributed to the meat yield and fatness index of the saltwater hard clam *Meretrix meretrix*. A new strain of *M*. *meretrix* with red shell color was produced after five generations of selection within a family of full-sibs. 7 morphometric traits, including shell length (*SL*), shell height (*SH*), shell width (*SW*), ligament length (*LL*), projection length (*PL*), projection width (*PW*), and live body weight (*LW*), and 2 meat characteristics, including meat yield (*MY*) and fatness index (*FI*) were measured from 50 individuals of three-year-old *M*. *meretrix*. The correlation coefficients, path coefficients, determination coefficients among attributes were analyzed. The results indicated that correlation achieved very significant levels (*P*<0.01). In addition, the multiple regression equations were formulated by considering the meat yield and fatness index as the dependent variables, respectively, and 7 other morphometric traits as independent variables. The correlation indices (*R*^*2*^) of morphometric traits against the meat yield and fatness index of clams were 0.901 and 0,929, respectively, indicating that the live body weight and shell length were the common main factors influencing the meat characteristics. By testing the significance of partial regression coefficient and gradually removing the non-significant morphometric traits, a multiple regression equation was established to estimate the relationship between shell length (*SL*, mm), live body weight (*LW*, g), ligament length (*LL*, mm) and meat yield (*MY*, %), fat index (*FI*, %): *MY* (%) = 0.432*SL*+0.251*LW* and *FI* (%) = 0.156*SL*+0.067*LL*+0.42*LW*-3.533. The study draws a conclusion that live body weight and shell length have a predominant direct effect on the meat yield and fatness index, which provides theoretical information for the breeding of *M*. *meretrix*.

## Introduction

The saltwater hard clam *Meretrix meretrix* (*M*. *meretrix*) is naturally distributed along the coastal areas of Asia and cultured widely in China because of its delicate flavor and high nutritional value [1, 2]. According to incomplete statistics, ten thousand tons of clams are annually exported to Japan from Nantong, China. As other bivalves such as oyster, Scallop, and Manila clam, *M*. *meretrix* presents a large polymorphism for shell colors and patterns, which can give consumers an aesthetic appreciation [3]. Furthermore, the red shell color of the clam is likely to attract the attention of domestic consumers, as the red color is associated with good luck and happiness in China [4]. We have found that the red shell color of the clams occurs in only 4% of the wild populations, making it potentially valuable for large-scale commercial production for the Chinese market. As a result, our group has produced a new strain of *M*. *meretrix* (Su haihong No. 1) with red shell color though five successive generations of selective breeding since 2007 [5].

The meat yield and fatness index of shellfish are closely related to processing and export, and are also important indicators to measure the quality of shellfish varieties [6, 7]. For bivalves, the meat yield is the ratio of meat weight to the weight of the shell immediately after slaughter, and fitness index is the ratio of meat weight to the weight of the shell after drying [8]. However, for a long time, the main indicators of breeding and improvement of bivalves focused on shell color and stress tolerance (e.g. low salt resistance, high temperature resistance, vibrio resistance, etc.) [9–11], while the research on meat characteristics such as meat yield and fatness index were relatively lacking. In bivalve breeding, breeding cannot be done by direct determination of meat production and fatness, because the bivalves has been killed and cannot be used as a parent for the next generation, even if it has high meat production or fatness. Therefore, it is necessary to find a more reasonable way to evaluate meat yields and fatness indices. The morphometric traits of clams can directly describe the shape of the clams, which has been shown to correlate with meat yield and fatness index. Consequently, the meat yield and fatness index of clams can be well estimated by morphometric measurements. Currently, the relationship between morphometric traits and meat production in tilapia *Oreochromis niloticus* and prawn *Litopenaeus vannamei* has been studied using path analysis, principal component analysis, or discriminative analysis, but has not been reported for clams [6, 12].

In this study, we explore the relationship between morphometric traits and meat characteristics by path analysis using the new strain of *M*. *meretrix* with red shell color as an experimental subject. The data obtained provide valuable information for 7 measurable traits that influence the meat characteristics and thus find a practical index to design of a reasonable breeding strategy.

## Materials and methods

### Experimental materials and preparations

**Fig 1:**
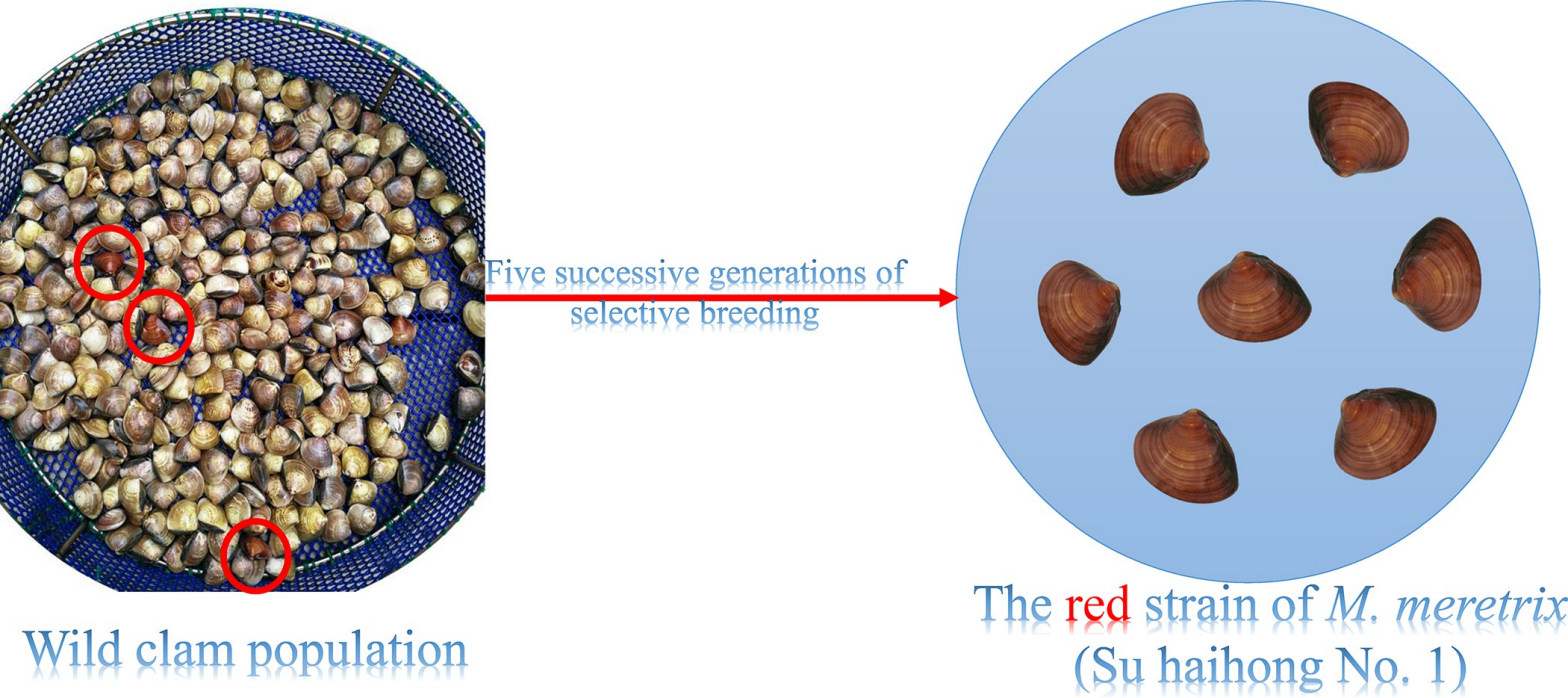
The red strain of saltwater clam *Meretrix meretrix*.

The red strain of saltwater clam *M*. *meretrix* that had been selected for five generations (Fig 1) were collected from the Jiangsu provincial fine breeds farm of Meretrix, Nantong, China. Before the experiment, all clams were washed and starved for a day in sand-filtered seawater. A total of 50 adult clams (3-year age) in August were selected randomly for the following experiments.

### Morphometric measurement of traits

**Fig 2:**
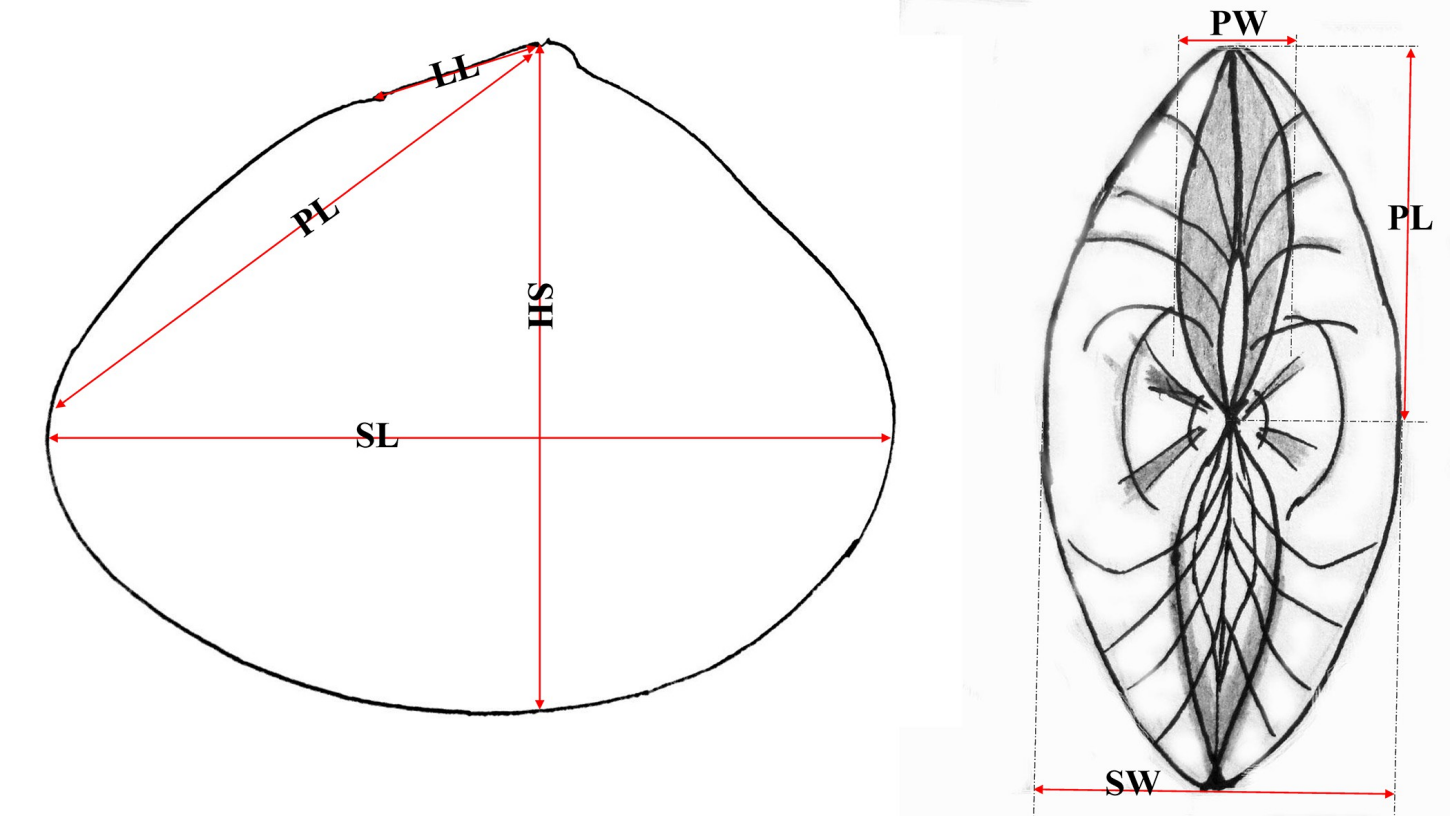
Morphological measurement of *Meretrix meretrix*.

The morphometric traits of clam (Fig 2), including the shell length (*SL*), shell height (*SH*), shell width (*SW*), ligament length (*LL*), projection length (*PL*), projection width (*PW*), and live body weight (*LW*) were individually measured using a vernier caliper (accuracy, 0.01 mm) and electronic balance (accuracy, 0.0001 g).

### Clam dissection and computation of meat characteristics

The cleaned clams were dissected to separate the edible muscle mass from the shell. The free moisture on the surface of the edible muscle and shell is then sucked up with absorbent paper, respectively. The wet weight of the edible muscle and shell was weighed by an electronic balance (accuracy, 0.0001g). Then, the edible muscle and shell was put in a thermostatic drier box at a constant temperature of 80°C for more than 12 hours and weighed by an electronic balance (accuracy, 0.0001g). The meat yield (*MY*) and fatness index (*FI*) were calculated using the following formula [8]:

$$Meat\;yield\;(MY)=\frac{\;wet\;muscle\;weight\;}{\;wet\;shell\;weight\;}\times 100\%$$

$$Fatness\;index\;(FI)=\frac{\;dry\;muscle\;weight\;}{\;dry\;shell\;weight\;}\times 100\%$$

### Data statistics and analysis

The mean values of the morphometric and meat characteristics, the standard deviation, and the coefficient of variation (CV) were calculated using Excel software (version 2022). The SPSS software (version 22.0) was used to evaluate the correlation coefficients between traits and to perform multivariate regression analysis, establishing the multivariate regression equations. Path coefficients, correlation indices, single-trait determination coefficients, and common-trait determination coefficients were derived using path analysis following the methodology published in previous studies [3].

## Results and discussion

### Statistical description of morphometric and meat characteristics

The statistical morphometric and meat characteristics of *M*. *meretrix*, as well as the coefficient of variation, are listed in Table 1, and the raw data for the measurements are shown in S1 Table. The results show that the average shell length (*SL*), shell height (*SH*), shell width (*SW*), ligament length (*LL*), projection length (*PL*), projection width (*PW*), and live body weight (*LW*) of 50 clams were (49.10±2.68)mm, (24.55±1.51)mm, (40.42±2.88)mm, (36.38±2.50)mm, (14.31±1.64)mm, (12.11±1.27)mm, and (31.3181±5.3659)g, respectively. The average meat yield (*MY*) and fatness index (*FI*) were (34.36±2.89)% and (6.33±0.74)%. Recent studies of the meat yield and fatness index of wild clams have reported meat yields of 32.14% and 6.16%, depending on individual size and gonad development cycle [7]. This value is lower than the one found in the red strain of *M*. *meretrix*, indicating that breeding was effective.

**Table 1:** Descriptive characteristics of the red strain of *Meretrix meretrix* (N = 50).

Characteristics		Mean	SD	(CV%)
**Morphometric characteristics**	***SL* (mm)**	49.10	2.68	5.46
	***SH* (mm)**	24.55	1.51	6.13
	***SW* (mm)**	40.42	2.88	7.12
	***LL* (mm)**	36.38	2.50	6.88
	***PL* (mm)**	14.31	1.64	11.44
	***PW* (mm)**	12.11	1.27	10.50
	***LW* (g)**	31.3181	5.3659	20.33
**Meat characteristics**	***MY* (%)**	34.36	2.89	8.40
	***FI* (%)**	6.33	0.74	11.76

### Correlation coefficients between the morphometric and meat characteristics

**Fig 3:**
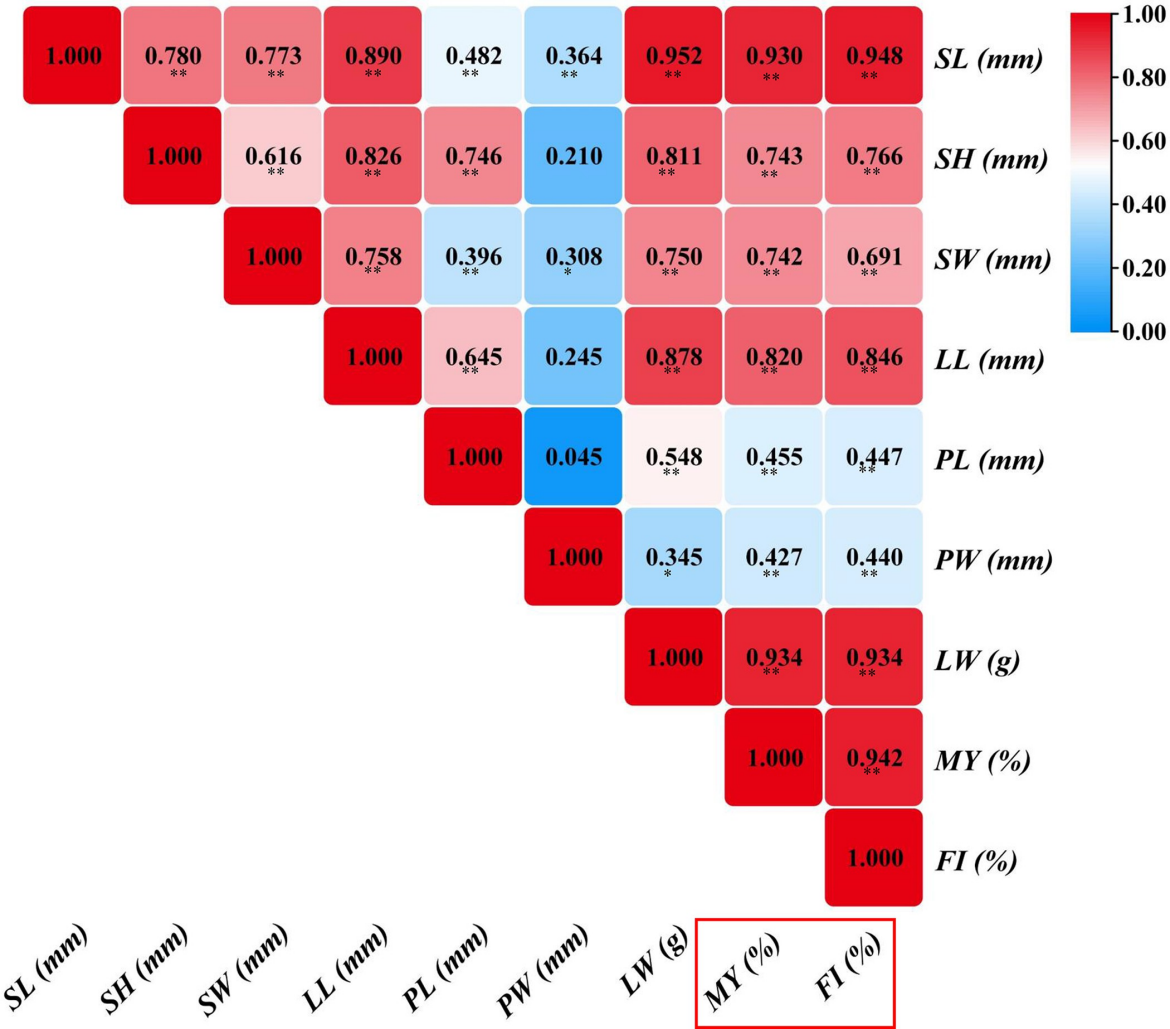
Correlation coefficients between the morphometric and meat characteristics of the red strain of *Meretrix meretrix* (N = 50). The closer it is to 1, the darker the red. The asterisk (*) and (**) indicate a significant difference at *P* < 0.05 and *P* < 0.01, respectively.

The correlation coefficients of each trait are presented in the heatmap (Fig 3), the correlation coefficients are depicted according to the color scale, and the closer it is to 1, the darker the red. The meat characteristic (*MY* or *FI*) is significantly positively correlated with all morphometric traits (*P*<0.01). Based on this, the correlation coefficient can be used as a condition for path analysis.

### Path analysis

The path coefficients and correlation indices were given in Tables 2 and 3. Path analysis was performed using morphometric traits including shell length, shell height, shell width, ligament length, projection length, projection width, and live body weight as independent variables; meat yield and fatness index as dependent variables, respectively. The correlation indices of the relationships between the morphometric traits and meat characteristics including meat yield and fatness index were decomposed to the direct effects through each trait and the indirect effects through other traits. The results showed that the live body weight (*P* < 0.01) and shell length (*P* < 0.05) significantly directly affected meat yield (Table 2). Additionally, the shell length (*P* < 0.01), shell height (*P* < 0.05), ligament length (*P* < 0.05), and live body weight (*P* < 0.05) significantly directly affected fatness index (Table 3). The ligament length and shell width indirectly correlated with the meat yield (Table 2), and the ligament length and shell height indirectly correlated with the fatness index (Table 3).

**Table 2:** Effects of morphometric traits on the meat yield of *Meretrix meretrix* (N = 50).

Traits	Correlation coefficients	Direct effect	Indirect effect							
			Total ∑	*SL* (mm)	*SH* (mm)	*SW* (mm)	*LL* (mm)	*PL* (mm)	*PW* (mm)	*LW* (g)
***SL* (mm)**	0.930**	0.401*	0.5284		0.0139	0.0366	-0.0703	-0.0134	0.0335	0.5281
***SH* (mm)**	0.743**	0.018	0.7254	0.3130		0.0292	-0.0652	-0.0208	0.0193	0.4499
***SW* (mm)**	0.742**	0.047	0.6947	0.3102	0.0109		-0.0599	-0.0110	0.0284	0.4161
***LL* (mm)**	0.820**	-0.079	0.8994	0.3572	0.0147	0.0359		-0.0180	0.0226	0.4871
***PL* (mm)**	0.455**	-0.028	0.4827	0.1934	0.0133	0.0188	-0.0509		0.0041	0.3040
***PW* (mm)**	0.427**	0.092	0.3352	0.1461	0.0037	0.0146	-0.0193	-0.0013		0.1914
***LW* (g)**	0.934**	0.555**	0.3791	0.3820	0.0144	0.0355	-0.0693	-0.0153	0.0318	

The asterisk (**) indicate a significant difference at *P* < 0.01.

**Table 3:** Effects of morphometric traits on the fatness index of *Meretrix meretrix* (N = 50).

Traits	Correlation coefficients	Direct effect	Indirect effect							
			Total ∑	*SL* (mm)	*SH* (mm)	*SW* (mm)	*LL* (mm)	*PL* (mm)	*PW* (mm)	*LW* (g)
***SL* (mm)**	0.948**	0.562*^%^	0.3858		0.0767	0.0366	0.0834	-0.0502	0.0419	0.3388
***SH* (mm)**	0.766**	0.098	0.6672	0.4382		0.0292	0.0774	-0.0777	0.0242	0.2886
***SW* (mm)**	0.691**	-0.136	0.8270	0.4343	0.0606		0.0711	-0.0412	0.0354	0.2669
***LL* (mm)**	0.846**	0.094*	0.7519	0.5000	0.0812	0.0359		-0.0672	0.0282	0.3124
***PL* (mm)**	0.447**	-0.104	0.5511	0.2708	0.0734	0.0188	0.0605		0.0052	0.1950
***PW* (mm)**	0.440**	0.115	0.3244	0.2045	0.0207	0.0146	0.0230	-0.0047		0.1228
***LW* (g)**	0.934**	0.356*	0.5778	0.5778	0.0798	0.0355	0.0823	-0.0571	0.0397	

The asterisk (**) indicate a significant difference at *P* < 0.01.

The determination coefficients of the morphometric measurements relative to the meat yield and fatness index were listed in Tables 4 and 5. The determination coefficients of individual morphometric traits relative to the meat yield and fatness index were on the diagonal. The results showed that the determination coefficient of the live body weight (0.3078) relative to the meat yield, while the determination coefficient of shell length (0.3156) relative to the fatness index was the largest. The co-determination coefficients of the morphometric traits against the meat yield and fatness index were above the diagonal. The results showed that co-determination coefficient of the live body weight relative to the meat yield (0.4239) and fatness index (0.3807) was the largest.

**Table 4:** Determinant and co-determinant coefficients of the morphometric traits on the meat yield of *Meretrix meretrix* (N = 50).

Traits	*SL* (mm)	*SH* (mm)	*SW* (mm)	*LL* (mm)	*PL* (mm)	*PW* (mm)	*LW* (g)
***SL* (mm)**	0.1610	0.0111	0.0294	-0.0564	-0.0108	0.0269	0.4239
***SH* (mm)**		0.0003	0.0010	-0.0023	-0.0007	0.0007	0.0160
***SW* (mm)**			0.0022	-0.0057	-0.0010	0.0027	0.0394
***LL* (mm)**				0.0062	0.0028	-0.0036	-0.0769
***PL* (mm)**					0.0008	-0.0002	-0.0170
***PW* (mm)**						0.0085	0.0352
***LW* (g)**							0.3078

R^2^ = 0.901.

**Table 5:** Determinant and co-determinant coefficients of the morphometric traits on the fatness index of *Meretrix meretrix* (N = 50).

Traits	*SL* (mm)	*SH* (mm)	*SW* (mm)	*LL* (mm)	*PL* (mm)	*PW* (mm)	*LW* (g)
***SL* (mm)**	0.3156	0.0862	-0.1178	0.0938	-0.0564	0.0470	0.3807
***SH* (mm)**		0.0097	-0.0164	0.0152	-0.0153	0.0048	0.0568
***SW* (mm)**			0.0184	-0.0193	0.0112	-0.0096	-0.0724
***LL* (mm)**				0.0088	-0.0126	0.0053	0.0586
***PL* (mm)**					0.0108	-0.0011	-0.0406
***PW* (mm)**						0.0132	0.0282
***LW* (g)**							0.1266

R^2^ = 0.929.

It was found that of all morphometric traits, only shell length and live body weight had a correlation index greater than 0.85 with meat performance. Only when the sum of the independent determination coefficients of each variable and the two joint determination coefficients of the dependent variable is ≥ 0.85, it indicates that the main independent variable affecting the dependent variable has been found [3, 13]. By testing the significance of partial regression coefficient and gradually removing the non-significant morphometric traits, a multiple regression equation was established to estimate the relationship between shell length (mm), live body weight (g) and ligament length (mm) and meat yield (%) and fatness index (%): *MY* (%) = 0.432*SL*+0.251*LW* and *FI* (%) = 0.156*SL*+0.067*LL*+0.42*LW*-3.533. The correlation indices (R^2^) of the morphometric traits on the meat yield and fatness index were 0.901 and 0.929, respectively. The regression prediction indicates that there is no significant difference between the estimated and actual observed values. This suggests that Eq. can be readily and reliably applied to selective breeding of the red strain of *M*. *meretrix*.

## Conclusion

In summary, genetic correlations are significant between morphometric traits, including shell length, shell height, shell width, and meat characteristics (meat yield and fatness index). The live body weight could be used as the primary trait for selective breeding of *M*. *meretrix*, which could indirectly lead to a large improvement in the meat characteristics (meat yield and fatness index).

## Supporting information

S1 Table The raw data of the measurements. (XLSX)

## References

[1] Wen X, Chen A, Wu Y, Yang Y, Xu Y, Xia W, et al. Comparative evaluation of proximate compositions and taste attributes of three Asian hard clams (*Meretrix meretrix*) with different shell colors. International Journal of Food Properties. 2020; 23(1): 400–411.

[2] Wu Y, Chen S, Chen A, Li Q, Zhang Y, Cao Y, et al. Molecular cloning and expression analysis of CDK genes during selective and wild populations of juvenile *Meretrix meretrix*. Aquaculture Research, 2022; 53: 5445–5454. doi: 10.1111/are.16025

[3] Huo Z, Wu Y, Gao Z, Chu G, Yan X, Yang F. Effects of shell morphological traits on the weight trait of the orange strain of the manila clam. Acta Ecologica Sinica. 2017; 37(2): 75–78. doi: 10.1016/j.chnaes.2016.12.007

[4] Ye S, Chen Y. The research on the commercial value of China red symbol. Hundred Schools in Art. 2008; 105(7):128–129.

[5] Zhang Z, Wu Y, Zhang Y, Cao Y, Chen S, Tian Z, et al. Effects of adding EM bacteria and mechanical aeration on water quality, growth and antioxidant status of *Meretrix meretrix* and *Exopalaemon carinicauda* farmed in the clam–shrimp polyculture system. Aquaculture Research. 2022; 53(5): 1823–1832.

[6] Dong Z, Liang Z, Xu P, Ming J, Zhang P, Zhang S, et al. Correlation between fillet yield and body measurements in Nile tilapia *Oreochromis niloticus*. *Journal of Fishery Sciences of China*, 2010; 17(2): 212–217.

[7] Zhang Y, Chen A, Wu Y, Cao Y, Chen S, Zhang Z. Analysis of economic characters of the selected line of red shell colored *Meretrix meretrix*. Journal of Aquaculture, 2022; 43(5): 1–5.

[8] Michael P, Crosby, Zhu G. Method for determining the condition index of bivalves. Journal of Foreign Aquaculture. 1992;04:34–35.

[9] Hu Z, Feng J, Song H, Zhou C, Wang M, Shi P, et al. Metabolic response of *Mercenaria mercenaria* under heat and hypoxia stress by widely targeted metabolomic approach. Science of the Total Environment. 2022; 809, 151172. 34710412 10.1016/j.scitotenv.2021.151172

[10] Liang X, Wang J, Liu Y, Peng L, Li Y, Batista F, et al. Complete genome of a marine bacterium Vibrio chagasii ECSMB14107 with the ability to infect mussels. Marine Genomics. 2019; 48, 100683.

[11] Yang Y, Ni J, Niu D, Zheng G, Li Y. Physiological response of the razor clam *Sinonovacula constricta* exposed to hyposalinity stress. *Aquaculture and Fisheries*. 2022;11002

[12] Zhang Q, Wang Q, Yu Y, Zhang C, Huang H, Li F, et al. Correlation of Morphometric Attributes to Net Weight and Fillet Yield of *Litopenaeus Vannamei*. Oceanologia et Limnologia Sinica. 2018; 49(3): 653–661.

[13] You W, Ke C, Luo X, Wang D. Genetic correlations to morphological traits of small abalone haliotis diversicolor. Journal of Shellfish Research. 2010; 29(3), 683–686.

